# Bovine mammary tissues are susceptible to infection by viruses bearing panzootic H5N1 influenza A virus glycoproteins

**DOI:** 10.1099/jgv.0.002338

**Published:** 2026-09-16

**Authors:** Rebecca A. Ross, Sarah K. Walsh, Hannah Montgomery, Hanting Chen, Edward Hutchinson, Pablo R. Murcia

**Affiliations:** 1 MRC-University of Glasgow Centre for Virus Research, Glasgow, UK

**Keywords:** bovine mammary gland, cross-species transmission, H5N1 subclade 2.3.4.4b, host range

## Abstract

The emergence of the panzootic clade 2.3.4.4b highly pathogenic H5N1 avian influenza in 2020 marked a major expansion in the host range of influenza A viruses (IAVs), raising concerns about further cross-species transmission events and zoonotic spillover. The introduction of 2.3.4.4b viruses into US dairy herds has resulted in widespread circulation, accompanied by reduced milk yield, mastitis and high viral loads in milk. Notably, virus circulation in dairy cattle represents a novel route for mammalian adaptation and transmission that has already led to more than 40 human cases in the USA since 2024. As haemagglutinin (HA) is the primary determinant of viral host range and tissue tropism, we investigated whether viruses containing the glycoproteins of avian H5N1 subclade 2.3.4.4b viruses could infect mammary tissue from Aberdeen Angus, Holstein Friesian and Limousin cattle. Using mammary gland explants, we inoculated tissues with attenuated reassortant viruses expressing the HA and neuraminidase (NA) of three 2.3.4.4b viruses that predated the emergence of H5N1 in the US cattle: A/chicken/England/053052/2021 (AIV07), A/chicken/Scotland/054477/2021 (AIV09) and A/chicken/England/085598/2022 (AIV48). Detection of viral NP antigen by immunohistochemistry in explants from both the teat and gland cistern for all three breeds following inoculation with AIV09 and AIV48 indicates that mammary tissue from each of the three tested cattle breeds is susceptible to infection by viruses bearing H5N1 glycoproteins. Lectin staining showed expression of both α2,3- and α2,6-linked sialic acids in the mammary tissue of all donors, indicating that all three breeds have the potential to support infection with both avian- and mammalian-adapted IAVs. Together, these findings demonstrate that the mammary glands from both beef and dairy cattle breeds are susceptible to infection with viruses bearing avian-origin H5N1 glycoproteins and suggest that bovine mammary tissue may provide an environment in which diverse IAVs could co-infect the same tissue. This possibility underscores the need for further investigation and the inclusion of both beef and dairy cattle in ongoing H5N1 surveillance and risk-assessment frameworks.

**Impact Statement**

The emergence of highly pathogenic H5N1 avian influenza in dairy cattle has expanded the recognized host range of influenza A viruses. Importantly, the ability of the virus to infect the mammary gland and transmit via milk revealed a novel interface for transmission to humans and animals. Although sustained circulation in US dairy herds has been reported, the susceptibility of mammary tissue from other breeds (including beef cattle) commonly used in different countries has been largely unexplored. Here, we show that viruses containing avian-origin H5N1 glycoproteins (haemagglutinin and neuraminidase) can establish infection in tissues derived from the mammary gland of three common cattle breeds (Aberdeen Angus, Holstein Friesian and Limousin). Virus was detected in epithelial cells from multiple breeds, indicating that viruses bearing H5N1 glycoproteins can infect tissues from both dairy and beef breeds. Receptor profiling showed abundant α2,3- and α2,6-linked sialic acids, consistent with a tissue environment that may support infection with both avian- and mammalian-adapted viruses. These findings demonstrate that both dairy and beef cattle breeds are susceptible to infection by viruses bearing H5N1 glycoproteins and strengthen the evidence base for including cattle in H5N1 surveillance and risk-assessment frameworks.

## Introduction

Goose/Guangdong-lineage (Gs/Gd) H5N1 highly pathogenic avian influenza (HPAI) viruses were first detected in 1996 in domestic geese in Guangdong, China, and quickly established themselves as a major cause of mortality in poultry and wild birds across Asia [1, 2]. Over subsequent years, long-distance migration and reassortment drove their global expansion, with detections across >80 countries by the mid-2010s [3]. A critical transition occurred with the rise of clade 2.3.4.4 viruses, which exhibit high genetic plasticity and frequent reassortment, yielding multiple H5N× constellations [3, 4]. Within this group, clade 2.3.4.4b became predominant from 2019 onwards, showing global dissemination and a substantial expansion in host range. This change in host range was characterized by extensive circulation in wild birds and repeated spillovers into a variety of mammalian species, including severe outbreaks in pinnipeds and mustelids [5–12]. Most recently, the detection of H5N1 infection in dairy cattle in the USA [13, 14] and the Netherlands [15] has underscored the threat of H5N1 spillover for both agriculture and public health.

Many factors influence the ability of influenza A viruses (IAVs) to infect different host species [16]. The primary determinant of IAV host range and tissue tropism is the glycoprotein haemagglutinin (HA), which mediates receptor binding and membrane fusion. IAVs primarily use α2,3-linked sialic acids in avian tissues and α2,6-linked sialic acids in human tissues. Other mammals exhibit varying balances of these receptors across tissue types, and these differences, alongside other host- and virus-specific factors, contribute to variation in IAV susceptibility across hosts and tissues. In cattle, infections with contemporary H5N1 viruses appear to show marked tissue tropism for the mammary gland [13, 17–20]. Affected cattle typically show mammary infection, with associated decreases in milk production, mastitis and high viral shedding in milk [13, 17, 18]. By contrast, reports of respiratory infection or respiratory signs remain limited [13, 18, 19, 21]. Observations of limited viral RNA in nasal swabs alongside high infectious titres in oral swabs and milk in affected herds support a model in which mammary infection and milk contamination play central roles in onward spread in dairy settings [22]. Sustained replication of H5N1 in the bovine mammary gland may therefore exert tissue-specific selection pressures that differ from those of the respiratory tract. The consequences of this selective environment for virus replication, transmission and cross-species infection remain unknown.

Here, we evaluated the susceptibility of mammary tissue from common beef and dairy cattle breeds to infection by viruses bearing the glycoproteins of avian-origin 2.3.4.4b H5N1 viruses to assess the potential for H5N1 glycoprotein-mediated infection in agricultural settings. We infected *ex vivo* cultures of mammary glands from six donors with attenuated reassortant viruses expressing the HA and neuraminidase (NA) of the H5N1 viruses A/chicken/England/053052/2021 (AIV07), A/chicken/Scotland/054477/2021 (AIV09) and A/chicken/England/085598/2022 (AIV48), or the HA and NA of human seasonal viruses (A/Norway/3433/2018 H1N1 and A/Norway/3275/2018 H3N2), and identified infected cells by immunohistochemistry (IHC). We additionally characterized the distribution of α2,6- and α2,3-linked sialic acids in donor tissues using lectin staining.

## Methods

### Biosafety considerations

All experiments with attenuated reassortant viruses were approved by the local genetic modification safety committee at the University of Glasgow (GM223), and the Health and Safety Executive of the UK. All experiments were carried out in appropriate laboratories by trained personnel at biosafety level 2. To allow us to investigate virus tropism, all attenuated reassortant viruses used in this study were derived by reverse genetics using the internal genes (*PB2*, *PB1*, *PA*, *NP*, *M* and *NS*) of the laboratory-attenuated vaccine strain A/Puerto Rico/8/34 (H1N1) and the glycoproteins (HA and NA) of the virus of interest. Additionally, all HA genes from HPAI viruses had the polybasic cleavage site (a major contributor to viral pathogenicity) removed. Work with the attenuated reassortant viruses used in this study was physically segregated from all other work with replication-competent IAVs to prevent reassortment generating viruses with novel HA/NA combinations.

### Collection of bovine mammary tissue

Udder tissue derived from six female cattle (Table S1) was collected from commercially slaughtered cows free from any antibiotic treatment or mastitis. Cows were all >2 years old at the time of sampling and lactation status was provided as a binary variable (Table S1). More detailed information on specific age, lactation stage or parity was not available. The teat cistern (TC) and gland cistern (GC) were removed from each udder quarter (Fig. S1) and transferred into Dulbecco’s Modified Eagle Medium (DMEM, Gibco) supplemented with 2% penicillin–streptomycin (Gibco) and 2% amphotericin B (Gibco), hereafter referred to as mammary explant growth media. Using a 5 mm biopsy punch (Covetrus), tissue cores (explants) were prepared from each tissue type and stored in mammary explant growth media until infection.

### *Ex vivo* culture

Mammary explants were prepared and maintained in culture as previously described [23]. Briefly, explants were placed in 96-well plates and submerged in mammary explant growth media supplemented with 10% heat-inactivated FBS (Gibco). Plates were cultured overnight in a humidified incubator at 37 °C with 5% CO_2_.

### Explant RNA extractions and pan-IAV quantitative reverse transcription polymerase chain reaction

All tissue RNA extractions were performed using a modified version of the RNeasy Plus RNA Extraction Kit (Qiagen). In brief, two tissue samples for each unique combination of donor and anatomical location were placed in separate 1.5 ml cryovials alongside 600 ul of Buffer RLT (Qiagen) containing beta-mercaptoethanol (ThermoFisher) and one 7 mm stainless steel bead (Qiagen). The samples were homogenized using a Precellys 24 Tissue Homogeniser (Bertin Technologies) set to 4,000 r.p.m. for 90 s, a total of three times. The supernatant was then transferred to a gDNA Elimination Column (Qiagen), and the extraction proceeded as per the manufacturer’s instructions.

Following the RNA extraction, a pan-IAV quantitative reverse transcription polymerase chain reaction (RT-qPCR) was performed using a QuantStudio 3 Real-Time PCR system (Applied Biosystems) to ensure that the samples were not infected with IAV prior to the experimental infection. In brief, the v2 Nagy M-gene primers [24] were used alongside the QuantiNova Probe RT-PCR kit (Qiagen), with all conditions as specified by the manufacturer’s instructions except for the annealing temperature, which was changed to 58 °C. Two explants from each tissue type and donor were tested, with two technical replicates of each sample run per RT-qPCR plate. All plates were run with three NTCs and three linearized M-gene-positive controls. Samples were considered negative if no amplification curve was observed within 40 cycles. All tested samples were observed to be negative.

### Cells and viruses

To allow us to investigate viral tropism and entry, attenuated reassortant viruses containing the HA and NA of the virus of interest and the internal genes of PR8 were generated for a panel of avian-origin H5N1 viruses and human seasonal IAVs [25]. HA and NA plasmids for the H5N1 viruses were a kind gift from Professor Munir Iqbal (The Pirbright Institute). HA and NA plasmids for seasonal H1N1 (accession number EPI_ISL_391292) and H3N2 (accession number EPI_ISL_390020) viruses were synthesized by GeneArt. Attenuated reassortant viruses and wild-type A/Puerto Rico/8/34 (PR8) were generated in HEK293T cells using the pDUAL reverse genetics system (Table 1), as previously described [25]. Viruses were passaged at a low m.o.i. in Madin-Darby canine kidney (MDCK) cells in viral growth media consisting of DMEM (Gibco) with 1 µg ml^−1^ TPCK-treated trypsin (Thermo) until ~70–80% cytopathic effect (CPE) was observed. At this point, the supernatant was clarified by centrifugation and stored at −80 °C in small volume aliquots. Only frozen aliquots were used in experimental infections to avoid freeze-thaw changes to infectious titre. Viral titre was determined using two methods: (1) plaque assays to determine p.f.u. per millilitre (p.f.u. ml^−1^) [26] and (2) RT-qPCR to determine genome copies per millilitre (copies ml^−1^) (see RT-qPCR section).

**Table 1.** Viruses used in this study. For all strains indicated, reassortant viruses were produced with the HA and NA genes of the indicated viruses and the remaining genes from the PR8 strain. For avian influenza virus (AIV) strains, the polybasic cleavage site of HA was replaced by a monobasic cleavage site

Short name	Isolate ID	Subtype
PR8	A/Puerto Rico/8/34	H1N1
H1N1	A/Norway/3433/2018	H1N1
H3N2	A/Norway/3275/2018	H3N2
AIV07	A/chicken/England/053052/2021	H5N1
AIV09	A/chicken/Scotland/054477/2021	H5N1
AIV48	A/chicken/England/085598/2022	H5N1

### Phylogenetic inference

To show the evolutionary relationships between the H5N1 isolates used in this study and other H5N1 isolates, a maximum likelihood (ML) phylogeny was inferred. Additional isolates included: A/goose/Guangdong/1/1996 (EPI_ISL_2584399), A/dairy cow/Texas/24-008749-001-original/2024 (EPI_ISL_19014384), Human A/Texas/37/2024 (EPI_ISL_19027114), A/dairy cow/USA/002645-005/2025 (EPI_ISL_19716907) and A/British_Columbia/PHL-2032/2024 (EPI_ISL_19548836). The phylogeny was inferred using PhyML v3.3.20180621 implemented within Geneious. A General Time Reversible (GTR) substitution model with a gamma-distributed rate variation (+G, four categories) and a proportion of invariant sites (+I) was applied. Model parameters (transition/transversion ratio, gamma shape parameter and proportion of invariant sites) were estimated from the data, and tree topology, branch lengths and substitution rates were jointly optimized. Branch support was assessed using 1,000 non-parametric bootstrap replicates. The resulting unrooted tree was subsequently rooted using the A/goose/Guangdong/1/1996 H5N1 HA sequence as an outgroup.

### Experimental infections

Media were removed from the mammary explants prior to infection. Viruses were diluted to 1×10^9^ genome copies per ml (measured using the pan-IAV RT-qPCR assay), and 5 ul was used to infect the apical surface of each explant for a final dose of 5×10^6^ genome copies per infection. Virus inocula were standardized based on genome copies per ml to provide a consistent measure of input viral material independent of differences in virus-specific entry efficiencies in indicator cell lines [27, 28]. Plaque titres and p.f.u.: genome-copy ratios for all virus stocks are provided in Table S2. Although the p.f.u. in the inocula varied among virus stocks, these differences did not correspond to the observed patterns of NP antigen detection in the mammary explants.

Two explants were infected per donor for each virus. After 1 h of incubation, the explants were re-immersed in mammary growth media. Plates were cultured for 24 h in a humidified incubator at 37 °C with 5% CO_2_. Explants were then fixed in 10% formaldehyde at 4 °C for 24 h, dehydrated in 70% ethanol and embedded in paraffin wax to generate formalin-fixed and paraffin-embedded (FFPE) tissue blocks. Longitudinal sections of 2–3 µm were taken for IHC and to assess epithelial integrity by haematoxylin and eosin (H&E) staining. For H&E staining, sections were deparaffinized and rehydrated prior to staining.

### Immunohistochemistry

FFPE tissue blocks were cut into 2–3 µm sections as described above and mounted on glass slides before undergoing deparaffinization and rehydration [29]. For IHC, sections were antigen-retrieved in pH 6.0 citrate buffer solution at 90 °C for 30 min. Sections were washed three times in 1× PBS (Thermo Fisher). To block endogenous peroxidase activity, slides were incubated in PBS with 3% H_2_0_2_ solution (Thermo Fisher) at 4 °C for 20 min, washed three times with PBS and blocked in 1% BSA (Thermo Fisher) for 30 min at room temperature (RT). Sections were then incubated with DA183 sheep anti-IAV-NP antibody (European Veterinary Laboratory, Clone HB65) diluted 1 : 500 in PBS with 1% BSA overnight at 4 °C. Finally, sections were washed again before being incubated in Biotin-SP-conjugated AffiniPure donkey anti-sheep IgG (Jackson ImmunoResearch) diluted 1 : 500 in PBS with 1% BSA for 1 h at RT. Sections were incubated with extravidin peroxidase (Sigma-Aldrich) diluted 1 : 100 in PBS with 1% BSA for 1 h at RT. 3,3′-Diaminobenzidine (DAB, Merck) was used as a chromogen, and Mayer’s hemalum solution (Sigma-Aldrich) was used as a counterstain in all IHC experiments. Slides were subsequently dehydrated and mounted as previously described [29].

### Lectin staining

Tissue sections were prepared as described above. Antigen retrieval was performed by incubating sections in 0.01 M EDTA for 30 min at 95 °C before cooling to RT. Sections were then blocked for 30 min in PBS with 1% BSA and washed three times with PBS before incubating overnight at 4 °C with 20 µg ml^−1^ of either fluorescein-conjugated *Maackia amurensis lectin* or CY5-conjugated *Sambucus nigra lectin* (both Vector Laboratories) diluted in PBS with 1% BSA. Sections were treated with streptavidin conjugated with Alexa Fluor 488 (Invitrogen) and incubated for 1 h at 4 °C. Finally, slides were stained with Hoechst (Thermo Fisher) diluted 1 : 500 for 30 min and mounted with ProLong Gold Antifade (Invitrogen). Images were captured using a Zeiss LSM 710 microscope with Fast Airyscan at 63× magnification.

### Digital pathology

For IHC and H&E, slides were digitalized and scanned on the Zeiss Axioscan 7 using the Plan-Apochromat 40×/0.8 objective. Image processing was conducted using Zeiss ZEN software (v3.10), with uniform exposure, gain, brightness and contrast for each tissue type.

## Results

### Bovine mammary tissue displays α2,3- and α2,6-linked sialic acids

**Fig. 1.**
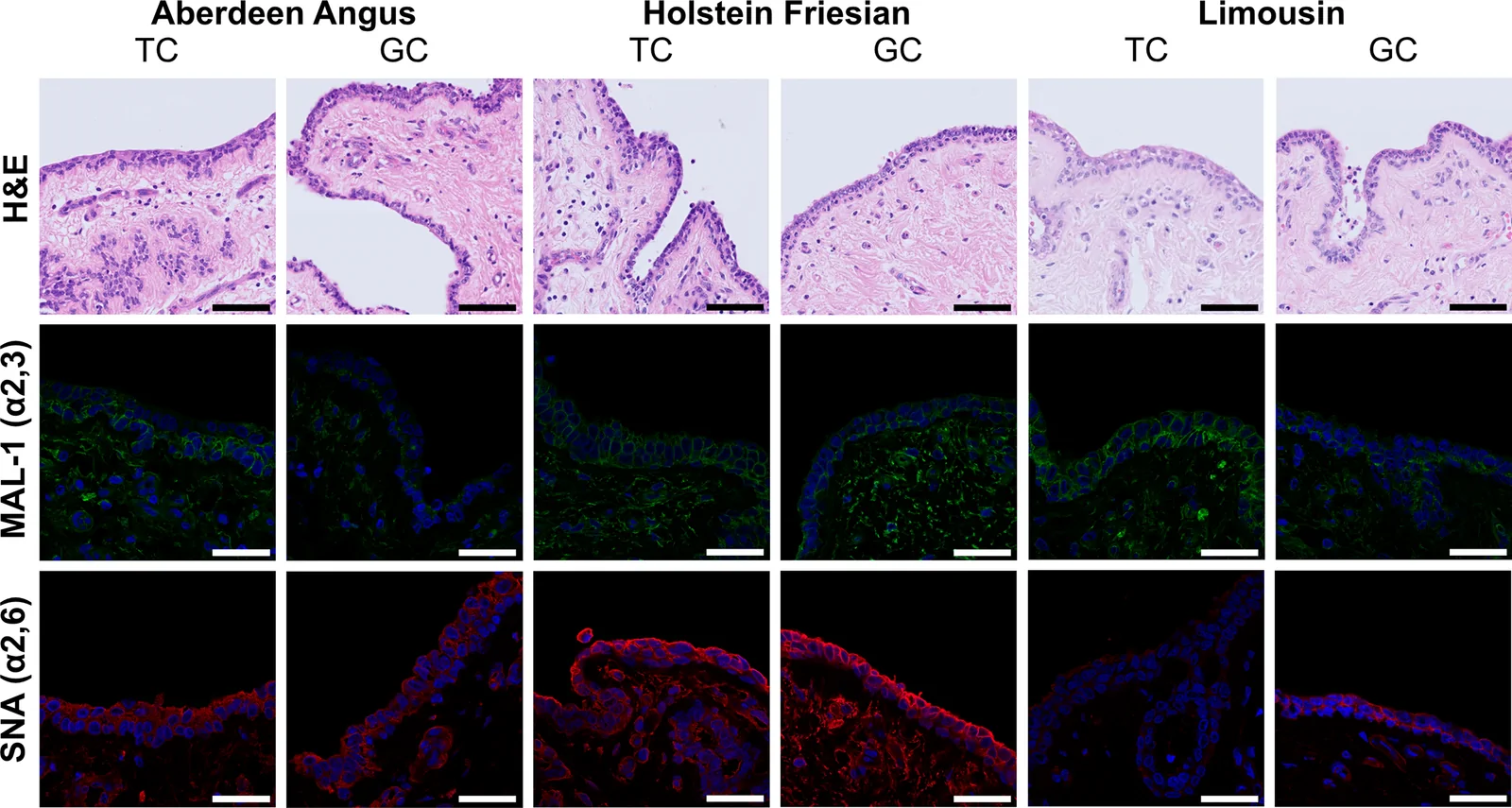
Lectin staining results for one representative donor of each cattle breed. The panels show H&E staining, MAL-1 staining for α2,3-linked sialic acids and SNA staining for α2,6-linked sialic acids for one donor of each breed for both the TC and GC. The scale bar on H&E panels represents 100 µm. For the lectin staining, α2,3-linked sialic acids are shown in green, α2,6-linked sialic acids in red and cell nuclei are shown in blue (DAPI-stained). Donor 2 is shown for Aberdeen Angus, donor 5 for Holstein Friesian and donor 4 for Limousin. The scale bar for lectin staining panels represents 100 µm.

To assess the display of IAV receptors in bovine mammary tissue, lectin staining was performed on FFPE blocks containing teat and gland cistern explants from Aberdeen Angus, Holstein Friesian and Limousin cattle. Staining with the lectins MAL-1 and SNA revealed widespread display of both α2,3- and α2,6-linked sialic acids in the epithelial cells of both the GC and TC across all three breeds (Fig. 1). This signal was predominantly localized to the luminal surface of epithelial cells, with the labelling presenting as multifocal to diffuse. No obvious qualitative differences in staining distribution or intensity were observed between the breeds or physiological states represented in this study. However, the limited and unbalanced donor set precludes robust assessment of breed- or lactation-associated differences, and substantial between-donor variation was observed within and across breeds (Table 2). These data demonstrate that bovine mammary tissue from both beef and dairy cattle breeds displays both α2,3- and α2,6-linked sialic acids.

**Table 2.** Lectin staining results for all donors

			Apical surface		Epithelial layer	
			α2,3	α2,6	α2,3	α2,6
AA	D1	Teat	+	++	+	++
		Gland	+	++	+	++
	D2	Teat	+	++	++	++
		Gland	+	++	++	++
	D6	Teat	+	++	+	++
		Gland	+	++	+	+
**L**	D3	Teat	++	++	++	+
		Gland	−	++	+	+
	D4	Teat	++	++	++	−
		Gland	++	++	++	+
HF	D5	Teat	+	++	+	++
		Gland	++	++	++	++

The table summarizes the localization of signals representing lectin staining results for each of the six donors used in this study. Samples are only reported as not showing expression if all biological and technical replicates of that unique combination of donor and tissue were negative. Cattle breeds are indicated with ‘AA’ for Aberdeen Angus, ‘L’ for Limousin and ‘HF’ for Holstein Friesian, individual donors are identified as D1–D6. Staining intensity was expressed by relative grades from negative (−) to weak (+) to strong (++), see Fig. S2 for representative images of each relative grade. MAL-1 = α2,3 and SNA = α2,6.

### Bovine mammary tissue is susceptible to infection with viruses bearing H5N1 clade 2.3.4.4b glycoproteins

**Fig. 2.**
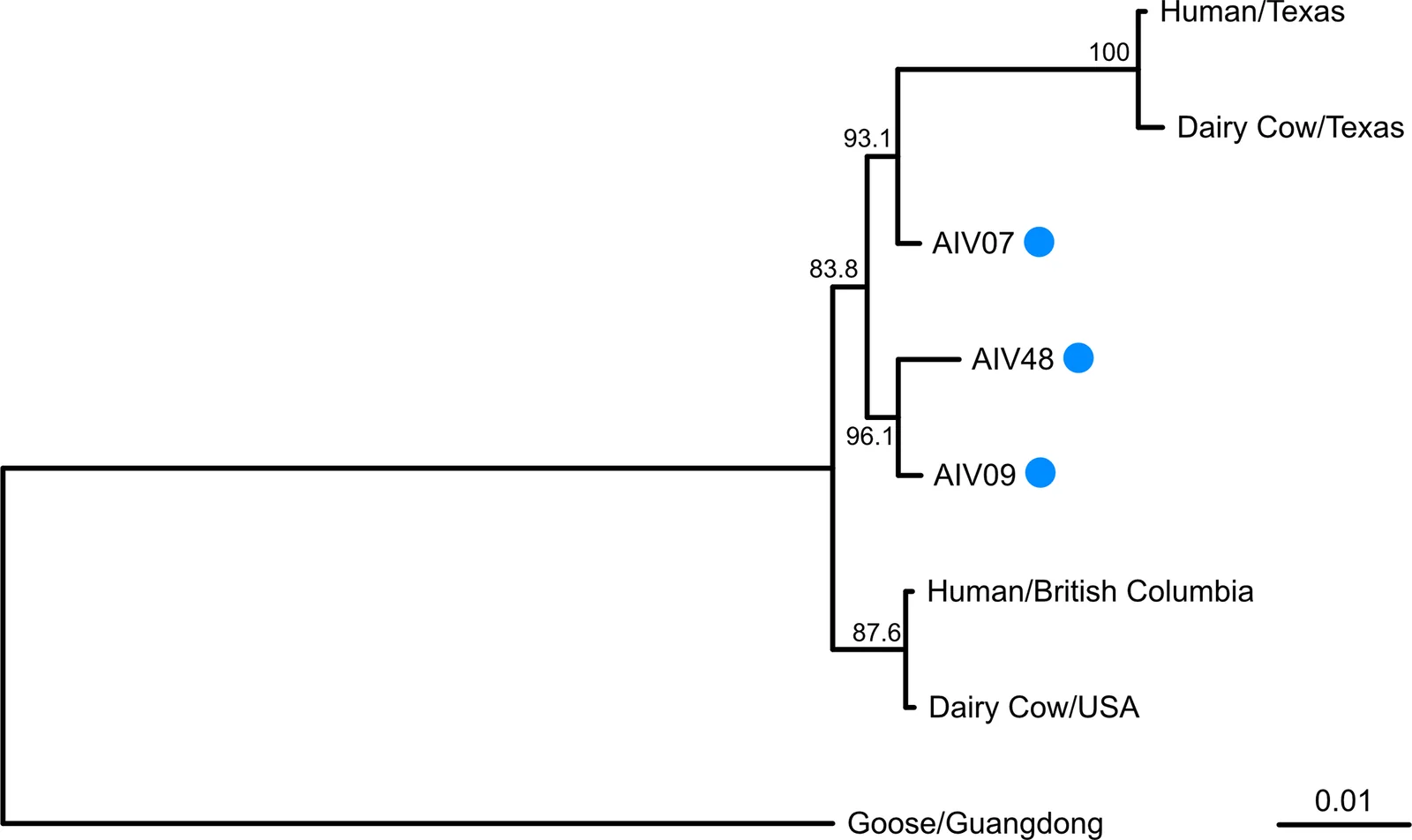
Phylogenetic tree of H5 genes used in this study. ML phylogeny of H5N1 HA sequences showing the evolutionary relationships between isolates used in this study and representative H5N1 viruses, including bovine and human isolates from the ongoing H5N1 epizootic (A/dairy cow/Texas/24-008749-001-original/2024, A/Texas/37/2024, A/dairy cow/USA/002645-005/2025 and A/British_Columbia/PHL-2032/2024) and a prototypic pre-2020 H5N1 isolate (A/goose/Guangdong/1/1996). The tree was inferred using PhyML in Geneious under a GTR+G+I substitution model with four gamma rate categories. Bootstrap values are displayed on the nodes, the scale bar indicates substitutions per site and blue circles denote viruses used in this study.

To determine whether bovine mammary tissue is susceptible to infection by viruses bearing avian-origin H5N1 glycoproteins, explants were inoculated with attenuated reassortant viruses bearing the HA and NA of three clade 2.3.4.4b H5N1 isolates (AIV07, AIV09 and AIV48; phylogenetic context shown in Fig. 2), alongside reassortants containing the HA and NA of human seasonal H1N1 and H3N2, wild-type PR8 and mock controls. After 24 h, the explants were fixed and stained for IAV nucleoprotein (NP) by IHC.

**Fig. 3.**
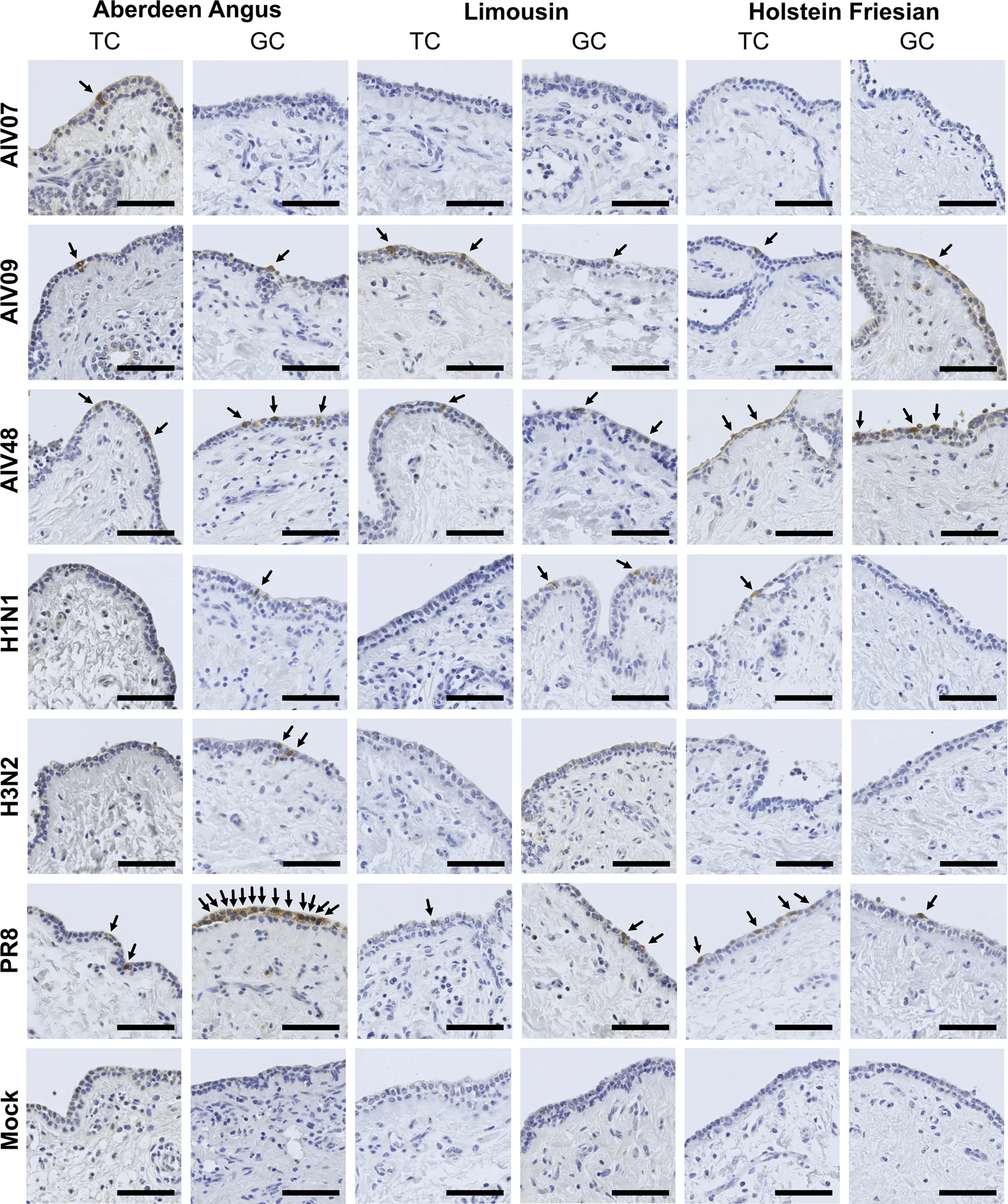
IHC staining results for one representative donor of each cattle breed. Each row represents tissue infected with one of six viruses or uninfected tissue (mock). Blue staining represents uninfected cell nuclei, and infected cells appear as brown and are highlighted with black arrows. Scale bars represent 50 µm.

NP antigen was detected in multiple explants across breeds, although the extent of NP-positive staining varied by virus, donor and tissue type (Fig. 3 and Table 3). Generally, donors displayed more NP-positive cells in the TC, with few NP-positive cells observed in the GC (D1–D4). Similarly, donors showed variation in the number of NP-positive cells across viruses, with all donors showing infection in at least one tissue when inoculated with AIV09, AIV48 or PR8. Infection with AIV07 was limited, with only the TC of D1 and D2 showing NP-positive cells. Only one donor (D2; Aberdeen Angus) showed NP-positive cells following inoculation with H3N2, and infection following H1N1 inoculation was limited, with only two donors showing NP-positive cells in the TC and three donors in the GC. D5, the only Holstein Friesian (dairy cow) in our study, showed extensive NP-positive staining following inoculation with both AIV09 and AIV48 in both the TC and GC. All donors from beef cattle breeds – Aberdeen Angus (D1, D2 and D6) and Limousin (D3 and D4) – had NP-positive cells for at least one virus in at least one tissue type. Within the examined donor set, we found no evidence of a consistent breed-restricted pattern of infection. However, the small and unbalanced donor set means that breed- and lactation-associated effects cannot be excluded. Overall, these findings indicate that bovine mammary tissue supports detectable NP antigen expression following inoculation with viruses bearing avian-origin clade 2.3.4.4b H5N1 glycoproteins, with the extent of NP-positive staining varying among donors and tissue sites.

**Table 3.** Susceptibility of cow teat and gland explants to infection with IAV

		Aberdeen Angus			Limousin		Holstein Friesian
		D1	D2	D6	D3	D4	D5
AIV07	Teat	Focal	Focal	–	–	–	–
	Gland	–	–	–	–	–	–
AIV09	Teat	Multifocal	Multifocal	Multifocal	–	Multifocal	Multifocal
	Gland	–	–	Focal	Focal	–	Multifocal
AIV48	Teat	Multifocal	Multifocal	–	Multifocal	–	Multifocal
	Gland	–	–	Multifocal/coalescing	–	Focal	Focal
H1N1	Teat	–	–	–	–	Multifocal	Focal
	Gland	–	Multifocal	Multifocal	–	Multifocal	–
H3N2	Teat	–	Multifocal	–	–	–	–
	Gland	–	Focal	–	–	–	–
PR8	Teat	Multifocal	Multifocal/coalescing	Diffuse	Multifocal/coalescing	–	Multifocal
	Gland	Diffuse	Multifocal	–	Multifocal	Multifocal/coalescing	Multifocal
Mock	Teat	–	–	–	–	–	–
	Gland	–	–	–	–	–	–

The table summarizes the extent and pattern of infection of each donor in this study with each of the six viruses used and a mock for both the TC and GC. Samples are reported as permissive (green box) if any infected cells were observed for all biological and technical replicates of that unique combination of donor, tissue and virus. See Fig. S3 for representative images of ‘focal’, ‘multifocal’, ‘multifocal/coalescing’ and ‘diffuse’ infection. Samples are only reported as non-permissive (grey box with ‘–’) if all biological and technical replicates of that unique combination of donor, tissue and virus were negative for infection.

## Discussion

The continued circulation of H5N1 in US dairy cattle, together with recent evidence of bovine exposure in the Netherlands, has raised concern about the risk of similar events in other countries with large cattle populations. This study aimed to assess the susceptibility of mammary tissue from three common cattle breeds to infection by viruses bearing avian-origin 2.3.4.4b H5N1 glycoproteins. NP antigen was detected in explants from both beef (Aberdeen Angus and Limousin) and dairy (Holstein Friesian) cattle, with no evidence for breed-restricted patterns of susceptibility. Lectin staining showed abundant α2,6-linked and α2,3-linked sialic acids in epithelial cells across all donors and breeds. Together, these findings indicate that both dairy and beef cattle breeds support infection by viruses bearing avian-origin H5N1 glycoproteins and may also be susceptible to IAVs with broader receptor usage.

Here, we have shown the co-expression of α2,3- and α2,6-linked sialic acids across three breeds of cattle. This is consistent with studies that have demonstrated widespread and heterogeneous expression of both α2,3- and α2,6-linked sialic acids in the bovine mammary gland [30–33]. The co-expression of α2,3- and α2,6-linked sialic acids is notable as receptor usage is a major determinant of IAV host range. While avian viruses classically prefer α2,3-linked receptors and human-adapted viruses more often use α2,6-linked receptors, recent work suggests that bovine-associated H5N1 viruses may show at least limited or dual engagement of both receptor types [20, 34, 35]. Sustained viral replication within the bovine mammary gland may consequently represent a distinct multicellular niche in which selective pressures differ from those operating in respiratory tissues [35, 36]. Even infrequent spillover events involving bovine mammary tissue may therefore provide opportunities for viral adaptation, potentially enabling avian-adapted viruses to increase their binding to α2,6-linked sialic acid receptors and improve their ability to infect humans. Further, work by Pinto *et al*. has demonstrated that bovine mammary cells support multi-cycle replication of diverse human and avian influenza viruses, including H1N1 and H3N2 strains, and can support the replication of reassortant viruses bearing H5N1 internal gene constellations [23]. Recent work has also shown that the efficiency of IAV replication in bovine cells varies considerably among viral genotypes and is strongly influenced by the internal gene constellation, highlighting that replication efficiency is not uniform across strains [26]. Together, these studies suggest that bovine mammary tissue may provide an environment that can support infection by IAVs of both avian and mammalian origin and could potentially facilitate co-infection. Whether this contributes to reassortment *in vivo* remains an important area for future investigation.

While large-scale outbreaks have thus far been reported primarily in dairy cattle in the USA, our data indicate that the mammary tissue of common beef cattle is also susceptible to infection by viruses bearing avian-origin H5N1 glycoproteins. All cattle breeds used in this study are classed as international breeds by the Food and Agriculture Organization of the United Nations, meaning they are used widely outside the UK, including in countries such as the USA, Australia, Germany and Argentina [37]. Given the broad geographic circulation of H5N1 in wild birds, including along migratory flyways, susceptible cattle populations in multiple regions may be at risk of exposure. Consequently, differences in outbreak magnitude between countries may be more plausibly explained by differences in population structure and management practices than by intrinsic biological resistance of particular cattle breeds. More broadly, these findings support the possibility that IAV infection in cattle is under-recognized. Historically, infection was regarded as very infrequent in cattle, but recent retrospective serology has identified NP-reactive antibodies in 586 of 1,724 bovine sera across multiple breeds and physiological states, suggesting that prior exposure may be more common than previously appreciated [38].

This study has several limitations that should be considered when interpreting the findings. First, the sample size was limited to six donors across three cattle breeds, with an unbalanced representation of production types. While the inclusion of both beef (Aberdeen Angus and Limousin) and dairy (Holstein Friesian) cattle did not reveal qualitative differences in receptor expression or susceptibility to infection by viruses bearing H5N1 glycoproteins, suggesting no strong evidence for systematic differences between beef and dairy cattle in this context, larger and more balanced studies will be required to fully resolve this question. Second, the use of attenuated 2 : 6 reassortant viruses with a shared PR8 internal gene cassette was specifically designed to investigate HA- and NA-mediated viral entry. As such, this system does not permit meaningful assessment of multi-cycle replication, which would be strongly influenced by heterologous internal genes rather than intrinsic properties of the H5N1 glycoproteins. Further investigation of wild-type H5N1 viruses would be needed to determine whether mammary tissue supports true multi-cycle replication. Third, the use of *ex vivo* mammary explants provides a controlled and physiologically relevant model of tissue infection but does not fully recapitulate *in vivo* conditions, lacking immune cell interactions, hormonal regulation, vascularization and intact tissue architecture, all of which may influence infection dynamics and permissiveness. Finally, limited information on host factors such as age, parity and stage of lactation further constrains interpretation, as these variables are known to influence mammary gland immune function and receptor biology, and may therefore impact susceptibility to infection [39–41].

Nonetheless, our findings show that mammary tissue from common cattle breeds is susceptible to infection by viruses bearing clade 2.3.4.4b H5N1 glycoproteins. These results support the view that bovine mammary epithelium is susceptible to infection by IAVs and raise concerns that IAV infection in cattle may be more widespread than previously assumed. Accordingly, cattle should be incorporated into H5N1 surveillance frameworks, particularly during poultry outbreaks or incursions of infected wild birds. Surveillance strategies that combine PCR-based detection of active infection with serology for prior exposure may provide a more complete picture of virus circulation at both individual- and herd-level. Overall, integrating cattle into preparedness planning is a precautionary and evidence-based response to the expanding host range of clade 2.3.4.4b H5N1 viruses.

## Supplementary material

10.1099/jgv.0.002338 Supplementary Material 1.

## Abbreviations

DAB: 3,3′-Diaminobenzidine
DMEM: Dulbecco’s Modified Eagle Medium
FFPE: formalin-fixed and paraffin-embedded
GC: gland cistern
Gs/Gd: Goose/Guangdong-lineage
GTR: General Time Reversible
HA: haemagglutinin
H&E: haematoxylin and eosin
HPAI: highly pathogenic avian influenza
IAV: influenza A viruses
IHC: immunohistochemistry
ML: maximum likelihood
NA: neuraminidase
NP: nucleoprotein
RT: room temperature
RT-qPCR: quantitative reverse transcription polymerase chain reaction
TC: teat cistern
