# Supplementary material for: Bovine mammary tissues are susceptible to infection by viruses bearing panzootic H5N1 influenza A virus glycoproteins
Source: J Gen Virol. 2026 Sep 16;107(9):002338. doi: 10.1099/jgv.0.002338 (PMC13581240; doi:10.1099/jgv.0.002338)
Supplement: Supplementary file 1 [file jgv-107-02338-s001.pdf]

Supplementary Data

Table S1: Table of breed, sex and lactation status of cattle.

Donor	Breed	Sex	Lactating (Y/N)
**1**	Aberdeen Angus	F	Y
**2**	Aberdeen Angus	F	Y
**3**	Limousin	F	N
**4**	Limousin	F	Y
**5**	Holstein-Friesian	F	N
**6**	Aberdeen Angus	F	Y

Table S2: Plaque forming units (PFU) and genome copies present in virus inocula.

	PFU/mL of original stock	Genome copies/mL of original stock	Ratio PFU:genome copies	Dilution factor to standardise to 1x10^{9} genome copies/mL	PFU in inocula	Genome copies in inocula
**AIV07**	8.00 x 10^{7}	9.69 x 10^{10}	0.0008	96.9	4.13 x 10^{3}	5.00 x 10^{6}
**AIV09**	3.30 x 10^{6}	8.65 x 10^{10}	0.00004	86.5	1.91 x 10^{2}	5.00 x 10^{6}
**AIV48**	2.70 x 10^{5}	1.19 x 10^{11}	0.000002	119.0	1.13 x 10^{1}	5.00 x 10^{6}
**H1N1**	1.00 x 10^{8}	3.46 x 10^{11}	0.0003	346.0	1.45 x 10^{3}	5.00 x 10^{6}
**H3N2**	1.00 x 10^{7}	8.33 x 10^{10}	0.0001	83.3	6 x 10^{2}	5.00 x 10^{6}
**PR8**	4.00 x 10^{7}	9.12 x 10^{10}	0.0004	91.2	2.19 x 10^{3}	5.00 x 10^{6}

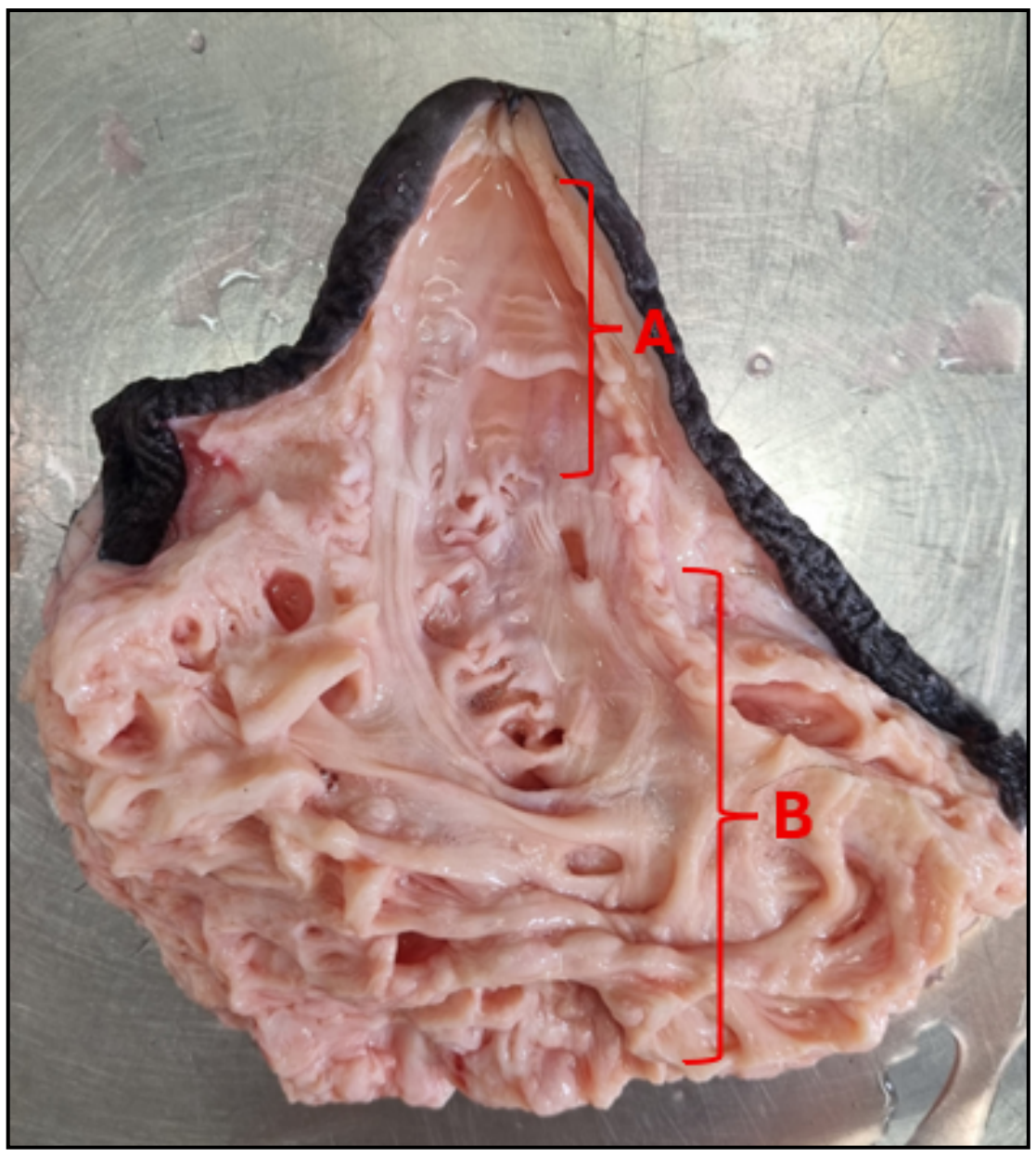

Figure S1: Sampling locations from the mammary glands. Cross section of teat cistern (A) and gland cistern (B) removed from mammary quarter.

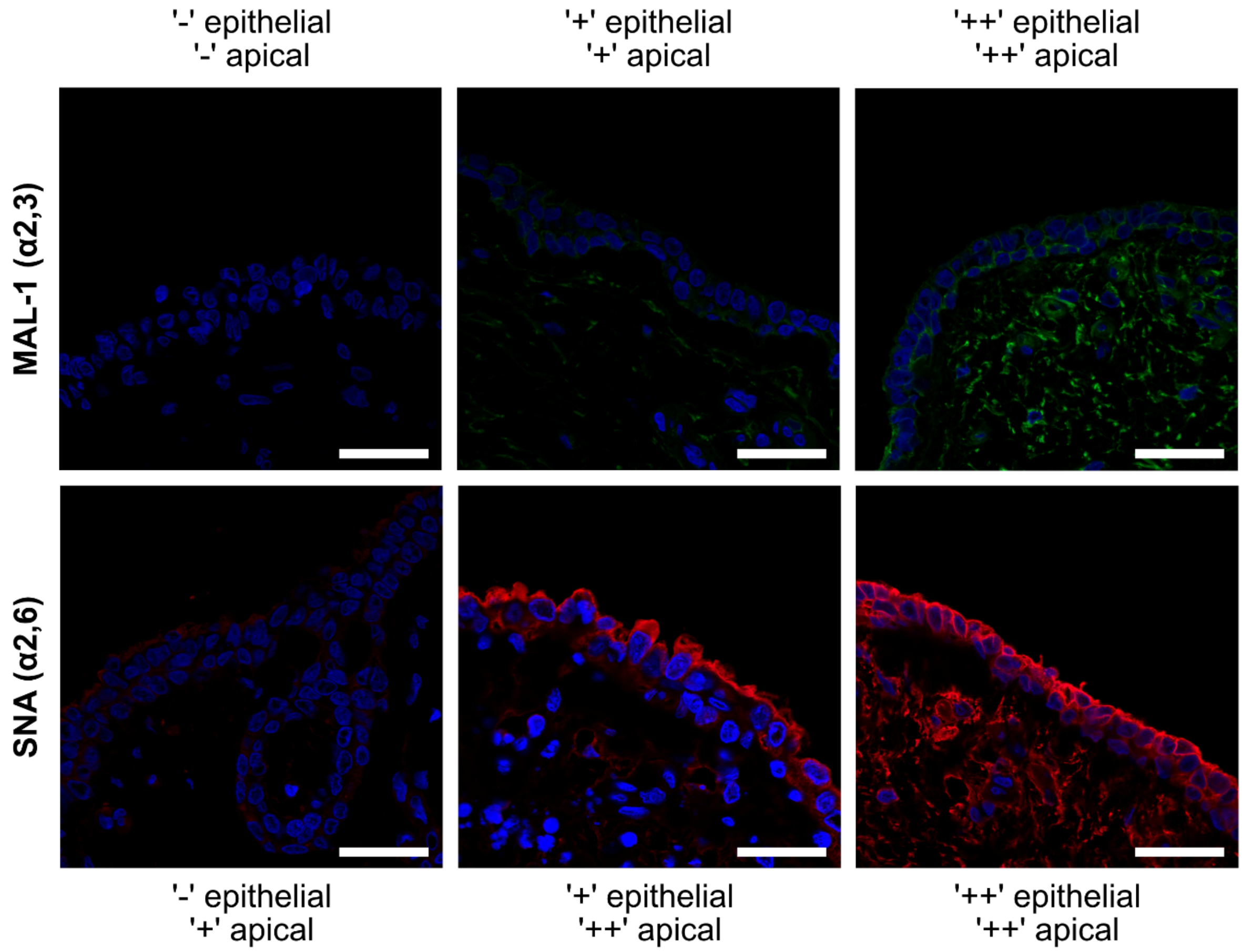

Figure S2: Representative images of each relative grade for the Lectin staining. Staining intensity was expressed by relative grades from negative (-) to weak (+) to strong (++). The above images show representative images of what each grade looked like. No captured samples were negative for SNA on the apical surface. MAL-1 = α2,3, SNA = α2,6. Scale bar represents 20μm.

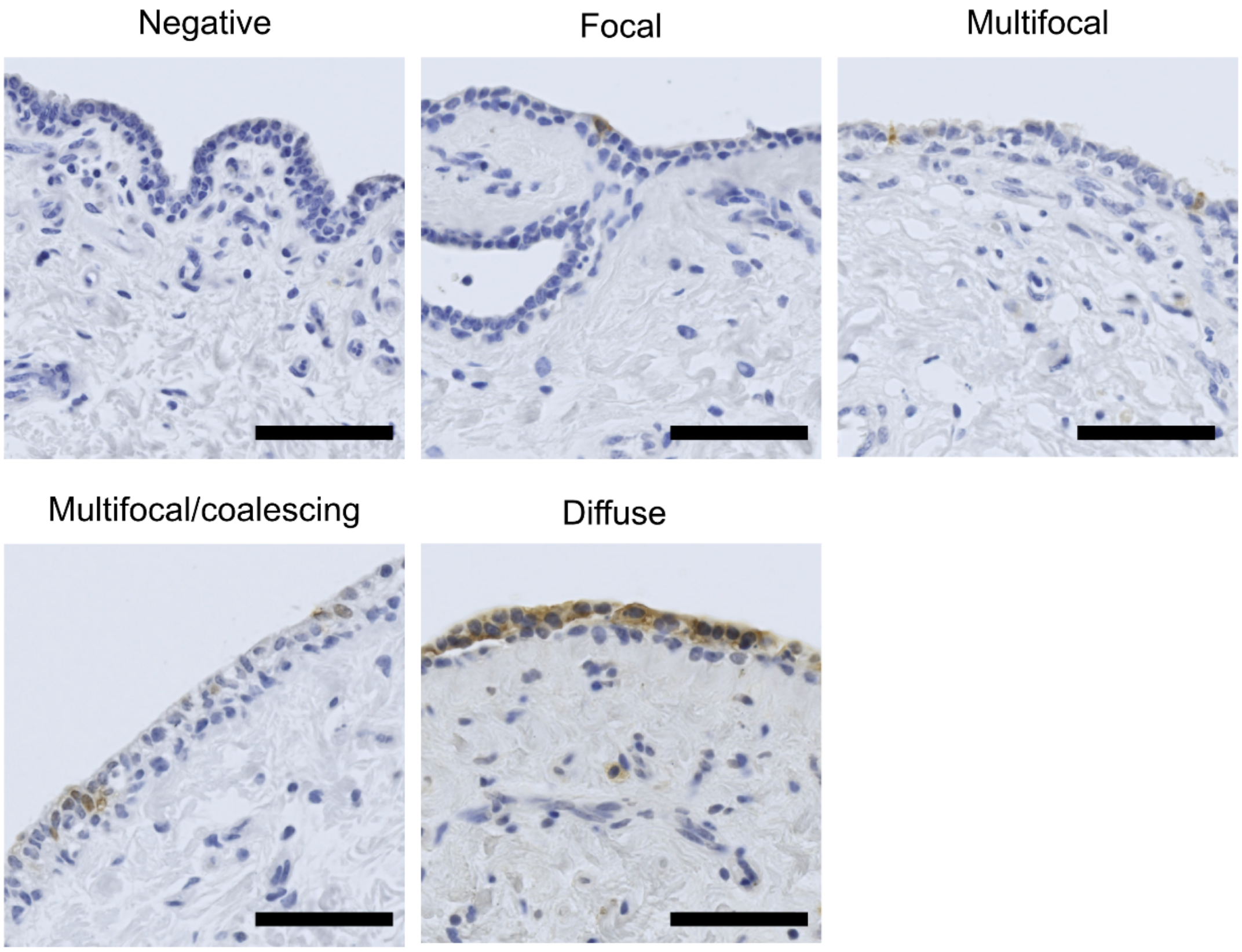

Figure S3: Types of infection reported in IHC staining. Representative images of each type of infection observed in the IHC staining: ‘negative’, ‘focal’, ‘multifocal’, ‘multifocal/coalescing’, and ‘diffuse’ infection. The scale bar represents 50μm.
